# Co-circulation and simultaneous co-infection of dengue, chikungunya, and zika viruses in patients with febrile syndrome at the Colombian-Venezuelan border

**DOI:** 10.1186/s12879-018-2976-1

**Published:** 2018-01-30

**Authors:** Marlen Yelitza Carrillo-Hernández, Julian Ruiz-Saenz, Lucy Jaimes Villamizar, Sergio Yebrail Gómez-Rangel, Marlen Martínez-Gutierrez

**Affiliations:** 1 Grupo de Investigación en Ciencias Animales-GRICA, Universidad Cooperativa de Colombia, Calle 30A #, 33-51 Bucaramanga, Colombia; 2 Universidad de Santander UDES, Facultad de Ciencias de la Salud, Programa de Bacteriología y Laboratorio clínico, Grupo de investigación en manejo clínico – CLINIUDES, Bucaramanga, Colombia; 3 Maestría en Investigación en Enfermedades Infecciosas, Universidad de Santander, Bucaramanga, Colombia; 4 Doctorado en Ciencias Básicas Biomedicas, Universidad de Antioquia, Medellin, Colombia; 5 Laboratorio Clínico, E.S.E. Jorge Cristo Sahium Hospital, Norte de Santander Cúcuta, Colombia

**Keywords:** Dengue virus, Chikungunya virus, Zika virus, Co-infection, Co-circulation

## Abstract

**Background:**

In Colombia, the dengue virus (DENV) has been endemic for decades, and with the recent entry of the chikungunya virus (CHIKV) (2014) and the Zika virus (ZIKV) (2015), health systems are overloaded because the diagnosis of these three diseases is based on clinical symptoms, and the three diseases share a symptomatology of febrile syndrome. Thus, the objective of this study was to use molecular methods to identify their co-circulation as well as the prevalence of co-infections, in a cohort of patients at the Colombian-Venezuelan border.

**Methods:**

A total of 157 serum samples from patients with febrile syndrome consistent with DENV were collected after informed consent and processed for the identification of DENV (conventional PCR and real-time PCR), CHIKV (conventional PCR), and ZIKV (real-time PCR). DENV-positive samples were serotyped, and some of those positive for DENV and CHIKV were sequenced.

**Results:**

Eighty-two patients were positive for one or more viruses: 33 (21.02%) for DENV, 47 (29.94%) for CHIKV, and 29 (18.47%) for ZIKV. The mean age range of the infected population was statistically higher in the patients infected with ZIKV (29.72 years) than in those infected with DENV or CHIKV (21.09 years). Both co-circulation and co-infection of these three viruses was found. The prevalence of DENV/CHIKV, DENV/ZIKV, and CHIKV/ZIKV co-infection was 7.64%, 6.37%, and 5.10%, with attack rates of 14.90, 12.42, and 9.93 cases per 100,000 inhabitants, respectively. Furthermore, three patients were found to be co-infected with all three viruses (prevalence of 1.91%), with an attack rate of 4.96 cases per 100,000 inhabitants.

**Conclusion:**

Our results demonstrate the simultaneous co-circulation of DENV, CHIKV, ZIKV and their co-infections at the Colombian-Venezuelan border. Moreover, it is necessary to improve the differential diagnosis in patients with acute febrile syndrome and to study the possible consequences of this epidemiological overview of the clinical outcomes of these diseases in endemic regions.

**Electronic supplementary material:**

The online version of this article (10.1186/s12879-018-2976-1) contains supplementary material, which is available to authorized users.

## Background

Viruses transmitted by arthropods (arboviruses) are the causative agents of some of the most significant infectious diseases emerging in the world. Among these arboviruses are the dengue virus (DENV), the chikungunya virus (CHIKV), and the Zika virus (ZIKV), which all circulate in the same tropical and subtropical geographical regions. These three arboviruses cause diseases with similar clinical presentations in the initial stages and in which the diagnoses are made clinically, particularly in countries where dengue is endemic. Dengue (the disease caused by DENV) is the most significant epidemiological arbovirus worldwide [1]. The term Dengue, includes both non-apparent or silent forms of the disease, the non-severe clinical forms (dengue fever [DF]) and severe clinical forms (dengue hemorrhagic fever [DHF] and dengue shock syndrome [DSS]). Although this classification of Dengue is still in force, the World Health Organization (WHO) proposed some years ago a new classification of the disease based on severity levels (severe and non-severe dengue). In the new classification, the non-severe dengue group includes patients who develop a self-limiting illness with fever, anorexia, nausea, rash, body aches, leucopenia and positive tourniquet test, and those patients who also have warning signs of developing severe dengue such as abdominal pain, persistent vomiting, fluid accumulation, mucosal bleeding, lethargy, liver enlargement and increased hematocrit with a rapid decrease in platelet count. The severe dengue group includes patients with severe plasma extravasation that can lead to hypovolemic shock or respiratory distress due to the accumulation of fluid in the lung, massive bleeding, and organ involvement (liver, heart or nervous system) [2]. An estimated 50 million cases of dengue fever occur annually, particularly in tropical and subtropical areas [3]. In turn, chikungunya fever (caused by CHIKV) and the disease caused by ZIKV have joined dengue as emerging arboviruses that threaten global public health [4, 5].

DENV and ZIKV belong to the family *Flaviviridae*, genus *Flavivirus*, whereas CHIKV belongs to the family *Togaviridae*, genus *Alphavirus*. All of these viruses possess a single-stranded, positive-sense RNA genome that encodes for both structural (forming part of the viral particle) and nonstructural (responsible for replication) proteins. The viral genome encodes three structural proteins, one of them being the envelope protein (E), which is the main inducer of neutralizing antibodies and is therefore responsible for classifying the virus into four serotypes: DENV-1, DENV-2, DENV-3, and DENV-4 [6].

DENV, CHIKV, and ZIKV are all transmitted by the mosquito vectors *Aedes aegypti* and *Aedes albopictus* [5, 7–9], causing clinical manifestations that are difficult to differentiate, including fever, myalgia, cephalalgia, arthralgia, conjunctivitis, and rash; thus, laboratory confirmation of cases is required [9]. Since to that these viruses are transmitted by the same vector, the co-circulation is frequent and, moreover, there is an increasing possibility of co-infection in those regions where the vector is present [10]. Additionally, the results of the analysis of the epidemics of CHIKV [11, 12] and ZIKV [13] in the Pacific, Asia, and more recently in the Americas have demonstrated an association between these viruses and adverse neurological, motor, and teratogenic events; even some authors have postulated that the clinical outcome could be affected with the co-circulation of the three agents [14, 15].

Colombia is considered a hyper-endemic country for DENV, registering more than 147,000 cases of dengue in 2010 [16]. The recent arrival of CHIKV in 2014 [17, 18] and the subsequent entry of ZIKV [19] into the country in 2015 have overwhelmed the Colombian health system [20] and have demonstrated the enormous need to improve the clinical definition of the diseases and reduce diagnostic errors [10]. Although the criteria for the clinical definition of patients with febrile syndrome because of DENV have been widely explored [7, 16], the lack of sufficient facilities to make an accurate viral diagnosis before the arrival of the new arboviruses created a diagnostic gap that has jeopardized the health of patients, even more so when the confirmation of cases, both of CHIKV and ZIKV, is performed by clinical definitions in regions that have already confirmed the circulation of these viruses [21, 22].

Furthermore, with the co-circulation of these three arboviruses (DENV, CHIKV, and ZIKV) in Colombia and different regions of Latin America, an increase of the number of reports of co-infections with different clinical outcomes was unusual [23–26]. However, to date, no information is available that establishes the prevalence of co-circulation and co-infection for these arboviruses in Colombia. Therefore, the objective of this study was to molecularly confirm the presence of DENV, CHIKV, and ZIKV and their co-infections in a cohort of patients with febrile syndrome consistent with dengue in the Colombian-Venezuelan region. Our results molecularly confirm the co-circulation of DENV, CHIKV, and ZIKV in equal dominance and with a significant prevalence of co-infection among them.

## Methods

### Study design

**Fig. 1:**
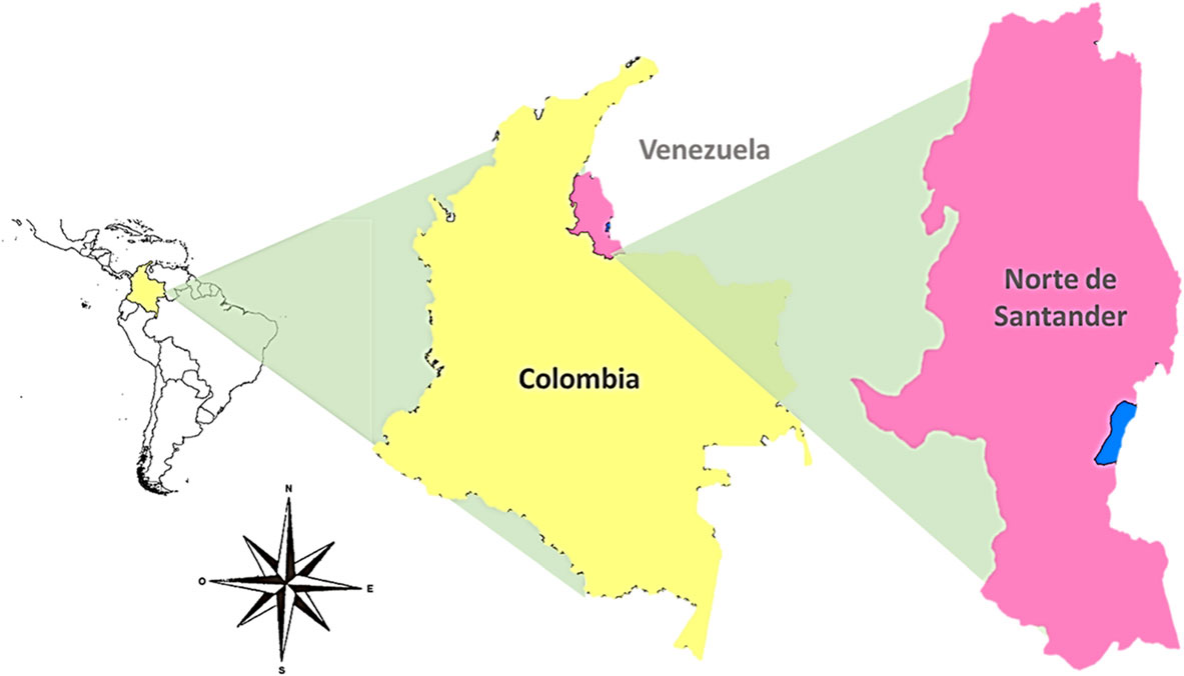
Geographical location of the sampling site in Colombia. The pink area denotes Norte de Santander Province, and the blue area denotes Villa del Rosario City. The map was created using DIVA-GIS software version 7.5.0 for Windows™. See text for references

An analytical cross-sectional study was developed in which serum samples were obtained from patients with febrile syndrome who consulted the emergency services of the Jorge Cristo Sahium Hospital in the municipality of Villa del Rosario, Norte de Santander, Colombia, between August 2015 and April 2016 (Fig. 1). This municipality is located at the northeastern Colombian-Venezuelan border. The study participants were people of any age, with a clinical diagnosis of febrile syndrome compatible with dengue, chikungunya fever, or zika fever, and in the acute stage of the disease, i.e., fever for no more than seven days.

### Ethical considerations

This study was approved by the Bioethics Committee of the Universidad Cooperativa de Colombia (Bucaramanga Headquarters) (Act from meeting April 16th of 2015) and by the technical committee of the E.S.E. Jorge Cristo Sahium Hospital (Rad # 087, July 28th, 2015). All persons 18 years and older, who met the clinical criteria signed the agreement or informed consent and completed a form for collecting general data, in which they were asked their age, sex, days of evolution of the fever, and possible previous diagnoses of DENV and/or CHIKV. In the case of children under 18 years old, the informed written consent was obtained from their parent or legal guardian. The samples and the data were analyzed anonymously.

### Taking samples and management of serums

A sample of 5 ml of venous blood was obtained from each patient in a dry tube. After 10 to 20 min, it was subjected to centrifugation for 5 min at 2500 rpm to obtain the serum. Subsequently, the serum samples were aliquoted and stored at − 70 °C until their respective use.

### RNA extraction and obtaining cDNA

All of the samples were subjected to RNA extraction with the Qiagen extraction kit (QIAamp® Viral RNA Mini Kit) according to the manufacturer’s instructions. The quality and quantity of RNA was determined by spectrophotometric analysis in a NanoDrop® 2000c UV-Vis spectrophotometer (Thermo Scientific®), and the quantified RNA was stored at − 70 °C until its use. The cDNA synthesis was performed with the RevertAid™ First Strand cDNA Synthesis Kit (Thermo Scientific®) following the manufacturer’s instructions, using a minimum of 0.5 μg of RNA and random primers for the retrotranscription. The cDNA was stored at − 70 °C until its use.

### Conventional PCR and real-time PCR for the identification of DENV

A conventional PCR was performed for the detection of the DENV genome, and the positive samples were confirmed by real-time PCR. In both cases, mD1 (F-5’-TCA ATA TGC TGA AAC GCG AGA GAA ACC G-3′) and D2 (F-5′-TTG CAC CAA CAG TCA ATG TCT TCA GG TTC-3′) primers were used for a region of the gene that encodes for the C-prM protein, which has been previously reported [27]. The amplification in the conventional PCR was performed with the Maxima® Hot Start Green PCR Master Mix (2X) enzyme (Thermo Scientific®), using 10 μmol of each primer and 2 μl of the cDNA from each sample. The amplification conditions included denaturation for 5 min at 95 °C, followed by 35 cycles at 95 °C for 30 s, 55 °C for 45 s, and 72 °C for 33 s, with a final extension at 72 °C for 10 min. To perform conventional PCR we used ProFlex 3 × 32-well PCR System (Thermo Fisher Scientific ®). The products were visualized on 1.5% agarose gel using SYBR® Safe (Thermo Scientific®) in a MiniBIS® (DNR Bio-Imaging Systems) gel photo documentation system. The expected size of the PCR products was 511 base pairs (bp). For the amplification in the real-time PCR, the Maxima® SYBR® Green/ROX qPCR Master Mix (2X) Kit (Thermo Scientific®) was used with the following thermal profile protocol: a first step of 50 °C for 2 min, a DNA polymerase activation step at 95 °C for 10 min, 35 cycles at 95 °C for 15 s for denaturation, 50 °C for 15 s for the alignment of the primers, and an extension at 72 °C for 30 s, measuring the fluorescence immediately after finishing the extension step. A melting curve (T_m_, melting temperature) was run after the final extension to confirm the specificity of the specific products. The melting curve included a step at 78.5 °C for 10 s and a ramp of 75 to 90 °C at a rate of 0.5 °C/s, with constant fluorescence measurements. To perform the real-time PCR we used the QuantStudio 3 Real-Time PCR System (Thermo Fisher Scientific ®) and the cut-off Ct value was ≤36, based in previous results of several groups, including ours, where both culture cells and serum specimens can be considered as positives when the value Ct was > 36 [28]. As positive control for all four serotypes of DENV we used supernatants of culture cells infected with reference strain of each virus obtained from the National Institute for Biological Standards and Control (DENV-1 West Pac 74, DENV-2 S16803, DENV-3 CH53489 and DENV-4 TVP360) and as negative control we used RNAse-free water.

### Multiplex PCR for serotyping of DENV

The samples that were positive for DENV by conventional PCR were processed for serotype identification by a multiplex PCR technique described previously [27]. Similarly, the Maxima® Hot Start Green PCR Master Mix (2X) enzyme (Thermo Scientific®) was used with 10 μmol of each of the following primers: MD1 (F-5’-TCA ATA TGC TGA AAC GCG AGA GAA ACC G-3′), rTS1 (F-5’-CCC GTA ACA CTT TGA TCG CT-3′), mTS2 (F-5’-CGC CAC AAG GGC CAT GAA CAG TTT-3′), TS3 (F-5′-TAA CAT GAG ACA GAG C-3′), and rTS4 (F-5’-TTC TCC CGT TCA GGA TGT TC-3′) and 2 μl of the cDNA from each of the samples. These primers amplify regions present in the gene that encode for the C-prM protein; the sizes expected for each amplified region were 208 bp (DENV-1), 119 bp (DENV-2), 288 bp (DENV-3), and 260 bp (DENV-4). The thermal profile for the amplification was the same as that described for the conventional PCR of DENV. To perform PCR we used ProFlex 3 × 32-well PCR System (Thermo Fisher Scientific ®). The products were visualized on 1.5% agarose gel stained with SYBR® Safe (Thermo Scientific®) under ultraviolet light using the MiniBIS® (DNR Bio-Imaging Systems) gel photo documentation system.

### Conventional PCR for the identification of CHIKV

A conventional PCR was also performed for the detection of the CHIKV genome. The Chik-1 (F-5′-TAA TGC TGA ACT CGG GGA CC-3′) and cChik-4 (F-5’-ACC TGC CAC ACC CAC CAT CGAC-3′) primers were used to amplify a region of the gene that encodes for the E protein, which has been reported previously [29]. The amplification was performed with the Maxima® Hot Start Green PCR Master Mix (2X) enzyme (Thermo Scientific®) using 10 μmol of each primer equally and 2 μl of the cDNA from each sample. The amplification conditions included the initial denaturation at 95 °C for 5 min, followed by 35 cycles at 94 °C (5 s), 64 °C (45 s), and 72 °C (30 s), with a final extension at 72 °C for 10 min. To perform PCR we used ProFlex 3 × 32-well PCR System (Thermo Fisher Scientific ®). The products were visualized on 1.5% agarose gel using SYBR® Safe (Thermo Scientific®) in the MiniBIS® (DNR Bio-Imaging Systems) gel photo documentation system. The expected size of the PCR products was 427 bp. As positive control of CHIKV we used supernatants of culture infected with a clinical isolate [30] and as negative control we used water RNAse free.

### Real-time PCR for the detection of ZIKV

Real-time PCR was used for the identification of ZIKV. The ZIKV1086 (F-5’-CCG CTG CCC AAC ACA AG-3′) and ZIKV1162c (F-5’-CCA CTA ACG TTC TTT TGC AGA CAT-3′) primers and the ZIKV1107-FAM (5’-FAM-AGC CTA CCT TGA CAA GCA GTC AGA CAC TCA A-3′) probe described previously [31] were used. The amplification was performed with the Taqman® Gene Expression Master Mix (Applied Biosystems) system, for which 5 μmol of each primer, 2.5 μmol of the ZIKV1107-FAM probe, and 2 μl of the cDNA from each sample were used. The amplification conditions were as follows: activation for 2 min at 50 °C and initial denaturation for 10 min at 95 °C, followed by 45 cycles at 95 °C (15 s) and 60 °C (60 s). To perform the real-time PCR we used the QuantStudio 3 Real-Time PCR System (Thermo Fisher Scientific ®) and the cut-off Ct value was ≤39 based on different criteria, among which are CDC interpretation criteria, considering a specimen positive if primer sets showed amplification with cycle threshold (CT) values ≤38.5 [31]. Different papers have showed that Zika virus could be tested positive in human and mosquito samples with CT values of 38–39 [32–34]; and a recent paper from Salvador, Bahia, Brazil, showed that serum samples with CTs > 34 could be confirmed positive for sequencing confirming that low viral copies are common in Zika virus positive patients [35, 36]. As positive control of ZIKV we used an isolated virus from *Aedes aegypti,* which was donated by the Programa de Estudio y Control de Enfermedades Tropicales (PECET) de la Universidad de Antioquia and as negative control we used RNAse free water.

### Genotypification of DENV and CHIKV by sequencing

The amplicons obtained by conventional PCR for DENV and CHIKV were purified and sequenced by Macrogen Inc. (Seoul, Korea) in an ABI 3730xl automated sequencer. To identify the DENV genotypes and CHIKV lineages circulating in the area during the study period, the sequences obtained were compared with various prototype strains of each of these viruses using BLAST and the on-line tool, Dengue, Zika & Chikungunya Viruses Typing Tool Version 0.9 – Alpha (available at www.bioafrica.net/software.php).

### Data analysis

The data were entered into a database in Excel. Subsequently, a descriptive analysis was performed of the included variables, estimating the measurements of frequency, central tendency, and dispersion for the quantitative variables and proportions for the qualitative variables. The prevalences were analyzed with the WinEpi on-line package (available at http://www.winepi.net) and were presented as absolute values and 95% confidence intervals (95% CIs). Comparisons between groups were performed using tests for parametric or nonparametric data according to the analysis of normality (Shapiro-Wilk normality test). The proportions were compared using the Chi-squared test. All statistical analyses were performed using Prism® 7.01 for Windows™ (GraphPad Software, San Diego, CA). In all cases, a *p value* < 0.05 was considered statistically significant.

## Results

### General description of the study cohort

Villa del Rosario is a municipality located in the department of Norte de Santander (Colombia) on the border with Venezuela; it is part of the urban area of the Metropolitan Area of Cúcuta. It has an altitude of 440 m above sea level and a population of 80,433 inhabitants, as reported in 2011 (Fig. 1). From August 2015 to April 2016, 157 serum samples were collected and evaluated in this study. The mean age of the patients was 26.81 years (SD ± 14.54 years); 103 (65.6%) were women and 54 (34.39%) were men. According to the data provided by the patients in the epidemiological survey, only eight (5.10%) reported ever having DENV infection and 19 (12.10%) reported having CHIKV infection.

Of the 157 patients involved in the study, 82 (52.2%) were positive for one or more of the arboviruses studied and 75 (47.8%) were negative. The mean age of the patients who were negative was 19.91 years (SEM ± 1.69 years), and that of the patients with confirmed infection was 22.6 years (SEM ± 1.62 years). In the group of positive patients, 25 (30.5%) were men and 57 (69.5%) were women, whereas in the group of negative patients, 29 (38.7%) were men and 46 (61.3%) were women, with no statistically significant differences between the two groups. The age group with the highest percentage of infection by one or more of the arboviruses evaluated was adults between 20 and 30 years old (28.0%), followed by children less than 10 years old (26.8%) and juveniles up to 20 years old (19.5%). Finally, the least affected groups were adults older than 30 years (Table 1).

**Table 1:** Distribution by sex and age of patients positive and negative for arboviruses

	Arbovirus negative (*N* = 75)	Arbovirus positive (*N* = 82)	*p*
Male % (N)	38.7 (29)	30.5 (25)	*0.281* ^*a*^
Female % (N)	61.3 (46)	69.5 (57)	
Age, Years (Mean ± SEM)	19.9 ± 1.7	22.6 ± 1.6	*0.245*
Year (s)			
0–10	33.3 (25)	26.8 (22)	*0.647*
11–20	25.3 (19)	19.5 (16)	*0.267*
21–30	21.3 (16)	28.0 (23)	*0.380*
31–40	8.0 (6)	11.0 (9)	*0.641*
41–50	6.7 (5)	7.3 (6)	0.001*
Older than 50	5.3 (4)	7.3 (6)	*0.953*

^*^p < 0.05 = statistically significant (Student’s *t*-test)

^*a*^Chi-squared (Χ^2^)

### Temporal distribution of infection by DENV, CHIKV, and ZIKV

**Fig. 2:**
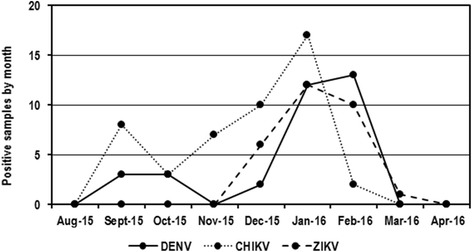
Temporal distribution of infection by DENV, CHIKV, and ZIKV. In general, the largest number of cases (70.7%) were reported between December 2015 and February 2016

As mentioned above, the samples were collected over a period of nine months; the highest number of cases (70.7%) during this period occurred between December 2015 and February 2016 (Fig. 2). For DENV, two peaks were evident: a smaller peak between the months of September and October 2015 (18.2% of the total cases of dengue) and a higher peak between the months of December 2015 and February 2016 (81.8% of the total cases). CHIKV displayed different behavior, with cases more evenly dispersed throughout a large part of the period under review (September 2015 to February 2016). Finally, the first cases of ZIKV were confirmed beginning in December 2015, and a number of cases continued to be sustained until the end of the period evaluated.

### Co-circulation of DENV, CHIKV, and ZIKV

The prevalence for DENV in the cohort of patients studied was 21.02% (95% CI: 14.65–27.39), which was confirmed by conventional PCR and real-time PCR; an attack rate of 40.99 cases per 100,000 inhabitants was also reported. The results of the serotyping by multiplex PCR showed that 30 samples (90.9%) belonged to DENV-2, two samples (6.1%) to DENV-1, and one sample (3.0%) to DENV-3. The mean age range of the infected population was 21.09 years (SEM 2.04), and women (72.7%) were more affected than men. The prevalence of CHIKV was 29.94% (95% CI: 22.77–37.10), which was confirmed by conventional PCR and sequencing; this corresponded to an attack rate of 58.38 cases per 100,000 inhabitants. The mean age of the infected population was 21.09 years (SEM 2.34), and again the most affected population was women, with 59.6% of the positive cases occurring in women. Finally, the prevalence of ZIKV, confirmed by real-time PCR, was 18.47% (95% CI: 12.40–24.54), corresponding to an attack rate of 36.02 cases per 100,000 inhabitants. Again, the population most affected was women (86.2%), but in contrast to what was found for DENV and CHIKV, the average age of the infected ZIKV population was statistically higher, with a mean of 29.72 years (SEM 2.09). The consolidated results of these analyses are shown in Table 2.

**Table 2:** Prevalence, attack rate, and distribution by sex and age of patients positive for DENV, CHIKV, and ZIKV

	DENV (*N* = 33)	CHIKV (*N* = 47)	ZIKV (*N* = 29)	*p*
Male % (N)	27.3 (9)	40.4 (19)	13.8 (4)	*n/a*
Female % (N)	72.7 (24)	59.6 (28)	86.2 (25)	*n/a*
Age, Years (Mean ± SEM)	21.1 ± 2.0	21.1 ± 2.3	29.7 ± 2.1*	0.006^a*^
Year (s)				
0–10	24.2 (8)	38.3 (18)	0 (0)	0.200^b^
11–20	24.2 (8)	14.9 (7)	20.7 (6)	0.366^a^
21–30	33.3 (11)	19.1 (9)	37.9 (11)	0.666^a^
31–40	12.1 (4)	10.6 (5)	24.1 (7)	0.838^a^
41–50	3.0 (1)	10.6 (5)	6.9 (2)	0.505^c^
Older than 50	3.0 (1)	6.4 (3)	10.3 (3)	0.337^c^
Laboratory diagnosis				
Prevalence % (95% CI)	21.0 (14.6–27.4)	29.9 (22.8–37.1%)	18.5 (12.4–24.5)	*0.462* ^*d*^
Attack rate × 100,000 Inhabitants	40.9 × 10^5^	58.4 × 10^5^	36.0 × 10^5^	*n/a*

*p < 0.05 = statistically significant

^*a*^Kruskal-Wallis test

^*b*^ *Unpaired t-test*

^*c*^ *One-way ANOVA*

^*d*^ *Chi-squared (Χ* ^*2*^ *)*

### Co-infection of DENV, CHIKV, and ZIKV

In addition to the co-circulation of the three studied arboviruses, co-infection was also found among them. The prevalence of DENV/CHIKV co-infection was 7.64% (95% CI: 3.49–11.80), that of DENV/ZIKV was 6.37% (95% CI: 2.55–10.19), and that of CHIKV/ZIKV was 5.10% (95% CI: 1.66–8.54), with attack rates of 14.90, 12.42, and 9.93 cases per 100,000 inhabitants, respectively. Furthermore, three patients were found co-infected with all three viruses (prevalence of 1.91%; 95% CI: 0.00–4.05), corresponding to an attack rate of 4.96 cases per 100,000 inhabitants. The mean age of the patients co-infected with either DENV/CHIKV, DENV/ZIKV, or CHIKV/ZIKV was 23.17 (SEM 4.57), 26.50 (SEM 2.35), and 30.13 (SEM 2.91) years, respectively, and that of the patients with the triple infection was 29.72 years (SEM 2.09). Except for the cases of DENV/CHIKV co-infection (in which both men and women were equally affected), women were more affected than men (with higher percentages reaching up to 87.5%). The consolidated results are shown in Table 3.

**Table 3:** Prevalence, attack rate, and distribution by sex and age of patients with co-infections

	DENV/CHIKV (*N* = 12)	DENV/ZIKV (*N* = 10)	CHIKV/ZIKV (*N* = 8)	DENV/CHIKV/ZIKV (*N* = 3)	*p*
Male % (N)	50.0 (6)	0 (0)	12.5 (1)	0 (0)	*n/a*
Female % (N)	50.0 (6)	100 (10)	87.5 (7)	100 (3)	*n/a*
Age, Years (mean ± SEM)	23.2 ± 4.6	26.5 ± 2.6	30.1 ± 2.9	30.3 ± 4.2	0.492^a^
Year (s)					
0–10	33.3 (4)	0 (0)	0 (0)	0 (0)	*n/a*
11–20	16.7 (2)	20.0 (2)	12.5 (1)	0 (0)	0.876^c^
21–30	16.7 (2)	50.0 (5)	25.0 (2)	33.3 (1)	0.475^b^
31–40	16.7 (2)	30.0 (3)	50.0 (4)	66.7 (2)	0.879^a^
41–50	8.3 (1)	0 (0)	12.5 (1)	0 (0)	*n/a*
Older than 50	8.3 (1)	0 (0)	0 (0)	0 (0)	*n/a*
Laboratory diagnosis					
Prevalence % (95% CI)	7.6 (3.5–11.8)	6.4 (2.6–10.2)	5.1 (1.6–8.5)	1.9 (0.0–4.1)	*0.205* ^*c*^
Attack rate × 100,000 Inhabitants	14.9 × 10^5^	12.4 × 10^5^	9.9 × 10^5^	4.9 × 10^5^	*n/a*

*p < 0.05 = statistically significant

^*a*^Kruskal-Wallis test

^*b*^ *One-way ANOVA*

^*c*^ *Chi-squared (Χ* ^*2*^ *)*

### Genotyping of co-circulating viruses

The analysis of the sequences performed using BLAST and the on-line DENV, ZIKV & CHIKV typing tool confirmed the circulation of the Asian/American genotype of DENV-2 with a homology of 99% with strains of DENV-2 previously reported in the region of Norte de Santander during 2005 (GQ868556). For DENV-3, the presence of genotype III displayed a 99% homology with strains previously reported in the north of Venezuela in 2007 (FJ898474). For DENV-1, the circulation of genotype V displayed a 98% homology with strains of DENV-1 previously reported in Colombia in 2008 (Santander - GQ868570) and in the Department of Sucre in 2009 (HM635907). The sequencing of CHIKV amplicons confirmed the presence of the Asian strain in Colombia and showed a 99% homology to different sequences previously reported from Colombia (KX496989, KU518343), Haiti (KX702402), Brazil (KU355832), Mexico (KT247385) and other countries from the Caribbean and Latin America. Neighbor-joining phylogenetic trees based on partial DENV and CHIKV Sequences are presented as Additional file 1: Figure S1, Additional file 2: Figure S2, Additional file 3: Figure S3, Additional file 4: Figure S4.

## Discussion

Our results regarding the simultaneous co-circulation of DENV, CHIKV, and ZIKV and their co-infections clearly demonstrate the critical epidemiological situation in the tropical and subtropical regions of the Americas and the increasing need to perform differential diagnosis in patients with acute febrile syndrome. All of the patients involved in our study met the basic criteria for dengue or acute febrile illness (temperature ≥ 37.8 °C for less than seven days; articular, retroocular, and/or extremities pain; rash; hemorrhage) [7], but despite this, only 52.2% of the patients could be diagnosed by molecular techniques for any of the three arboviruses analyzed (DENV, CHIKV, or ZIKV) and the 47.78% was negative. Acute febrile syndrome is a common feature of several infections endemic arbovirus countries and although Dengue in the most common cause of “Febrile Syndrome” in Colombia [37], other etiological agents, such as bacterial (Leptospira spp., Rickettsia spp., Salmonella spp. and Brucella spp) [38, 39], viral (influenza virus, hepatitis A virus, B and C, Venezuelan equine encephalitis virus, Mayaro virus and Hantavirus) [40] or parasitic (Plasmodium vivax, P. falciparum) [41], could be the cause for 47.78% of acute febrile syndromes. In ongoing studies we are using multiple approaches to try to explain and to reveal the etiological agent for those cases (data not shown). On the other hand, we believe that false negative results are less likely because all the participants enrolled had to be in acute stage of the disease, that is to say, fever no more than 7 days. Moreover, the samples were stored at the adequate temperature (− 70 °C) and the quality of RNA was confirmed by the ratio of absorbance at 260 nm and 280 nm (measured by spectrophotometry), being in all cases > 2.0.

As it can be observed in the Fig. 2, there was in increase in cases from December through February. This increase can be due to the arrival of ZIKV to Colombia, because the first case was reported in October 2015 and the higher peak of the outbreak was observed from December through February [19]. Moreover, the increase too can be due to those months which coincide with holiday season in Colombia when the people who live in regions with low temperature (where there is not presence of *Aedes*) move to regions with higher temperature (with presence of *Aedes*), increasing the possibilities of transmission of infection.

The prevalence of DENV in the population evaluated was 21.02%, with an attack rate of 41 cases per 100,000 population. These data are in stark contrast with other studies conducted in the country that have reported the presence of DENV in approximately 30% of patients with febrile syndrome [42] and a cumulative incidence of cases ranging between 36.5 and 268.7 cases/100,000 inhabitants in different regions of Colombia [43, 44]. This low DENV detection rate can be explained by an increase in the number of cases of the other arboviruses studied, as it has been previously reported in India, where the prevalence of DENV decreased with the increase in cases of CHIKV [45, 46].

As could be seen in Table 2, there is an age-related decline in DENV disease. This phenomenon has been widely reported for Colombia with the highest incidence in individuals 5–14 years of age [16]. This epidemiological behavior could be explained by the high endemicity of the country, on which individuals acquires DENV Infection at young age with the subsequent immunity. For example, in Medellín (City located in a central region of Colombia) the 70% of the younger people (up to 21 years old) have antibodies against DENV due to primary infections acquired in childhood [47]. This same endemic epidemiological status could be expected in the next years for CHIKV and ZIKV in several Colombian municipalities due to the similarity of DENV, ZIKV and CHIKV transmission cycle which bring on the possibility of endemic establishment of those arboviruses [48].

Although an end to the Chikungunya epidemic in Colombia had already been officially declared for the time period in which the sampling was performed, the prevalence obtained (29.94%) showed that CHIKV had been established as one of the most significant arboviruses in the diagnostic panel, possibly establishing endemic cycles [4]. Additionally, our data show an estimated 58.38 cases/100,000 inhabitants, which is considerably higher than the average attack rate established for Colombia during the epidemic period of 19.1 cases/100,000 inhabitants (range between 4.5 and 283.1) [49]. These figures are important because although it is well known that mortality due to CHIKV is considerably less than that occasioned by DENV [1], the chronic sequelae of CHIKV infection may have a greater long-term impact, affecting up to 25% of infected patients [12]. This chronic sequelae is mainly associated with “post-chikungunya chronic inflammatory rheumatism” and thus has a significant impact on the future quality of life of the population [11].

Our sampling began in the month of August 2015, a time period in which the entry of ZIKV into Colombia had not yet been reported. According to reports from the National Institute of Health [19], the first cases of ZIKV that were identified in our study occurred in the month of November 2015. The prevalence of ZIKV was found to reach 18.47%, with an attack rate of 36 cases/100,000 inhabitants, which is considerably lower than that reported for the entire department of Norte de Santander of 655 cases/100,000 inhabitants [19]. Notably, the official data represent the total number of patients diagnosed for a clinical picture compatible with infection by ZIKV whereas our results present the number of laboratory-confirmed cases, which typically are presented in lesser number [8]. These differences are a result of the difficulties in the molecular diagnosis of ZIKV because of the low viral loads present in the serum of patients [24, 36]. In agreement with the findings reported, in our study, the amplification “threshold cycle” of the qPCR was greater than 29.09.

Although it is not currently possible to speak of susceptibility by gender, our results are in complete agreement with various studies showing that the ratio of cases between women/men is generally at a ratio of 2/1 or 3/1 in DENV, CHIKV, or ZIKV infections. This relationship has been previously reported in Colombia for DENV infections [16], during the CHIKV outbreak [18], and recently for ZIKV [19], as well as being previously reported internationally during other events, both endemic in the case of DENV [50, 51] and epidemic in the case of CHIKV and ZIKV [52, 53]. This difference in the presentation of infection in women could be the result of socio-cultural practices that lead to greater medical consultation by women or more actual risk associated with the domestic behaviors of women and the domestic behavior of *A. aegypti* [54], which favor an increased risk of exposure.

Importantly, our study also found a higher prevalence of infection by ZIKV among women aged 20–40 years than has been reported for the rest of the country [19] and for other countries in the region during the current outbreak [53]. Taking into account the possible adverse effects associated with infection by ZIKV during pregnancy [55, 56], it is important to focus on prevention efforts in this age group to reduce unwanted pregnancies and to increase surveillance of such adverse events in pregnant women infected with ZIKV and their babies [57].

The co-circulation of the four serotypes of DENV has been previously reported in the country [16], and our results are consistent with those reports because we found the circulation of serotypes − 1, − 2, and − 3, although with a higher prevalence of DENV-2 (90.9%). However, the analysis of the sequenced samples demonstrated that the DENV-2 Asian/American genotype predominated, which is consistent with reports in recent years in the northern region of Colombia [16]. Genotype V was identified for DENV-1, which is also representative of most of the strains of this serotype isolated in Colombia and generally in the Americas [58, 59]. Finally, genotype III was found in the DENV-3 strains, which has been the predominant genotype in Colombia since its introduction in 2002 [60]. In the case of the samples positive for CHIKV, sequencing revealed the presence of the Asian strain, the same strain that has been previously reported in Colombia [18, 61, 62]. Finally, although no sequencing of the samples positive for ZIKV was performed because the real-time PCR primers amplify a specific region of the Asian/Caribbean strain [31], we can presume that these samples belong to this lineage, which is the same that has been previously reported in the country [62–64]. Subsequent phylogenetic analyses are necessary to understand the molecular epidemiology of DENV, as well as of CHIKV and ZIKV. In other studies, ongoing, we are evaluating the molecular epidemiology (by sequencing) of those positive samples to identify the evolution level and adaptation of those virus strains in the region.

The co-circulation of DENV, ZIKV, and CHIKV is a chance occurrence that has triggered alarms in all tropical and subtropical regions of the world, not only because of the difficulty of making an accurate clinical diagnosis, but because of the potential epidemiological effects of co-infections. Although some cases of co-infection among these arboviruses have recently been reported in Colombia [23, 25], our study is the first cross-sectional study that demonstrates the extent of the co-circulation and the possibilities of co-infection of these three arboviruses in Colombia, demonstrating that although there is an apparent dominance of infection by Chikungunya, the three agents co-circulate simultaneously in the evaluated region. A similar context has been previously described in India, where the simultaneous co-circulation of DENV, CHIKV, and ZIKV was demonstrated in equal proportions during 2010 [46] and subsequently during 2013 [65], eliminating the possibility of displacement of any of the viruses [66], as has been shown between different genotypes of DENV, both in the Americas [67] and in Asia [68].

The reported prevalence of DENV/CHIKV co-infection varied from very low (2.8%) [9] to more than 10% [65, 69, 70], which is consistent with the findings in our study (7.64%). However, although there are very few studies that present the prevalence of DENV/ZIKV or CHIKV/ZIKV co-infections, our results demonstrate a higher prevalence of DENV/ZIKV co-infection (6.37%) than that reported in Pernambuco, Brazil (2.6%) [26] or in Nicaragua (1.7%) [70] and a lower prevalence of CHIKV/ZIKV co-infection (5.10%) than that recently reported in Bahia, Brazil (13.3%) [24], but a similar prevalence of CHIKV/ZIKV to that reported in Nicaragua (4.6%) [70]. Finally, this is the first study that reports the prevalence of triple co-infection (1.9%) in South America, but the result is consistent with the recently reported prevalence in Central America (1.7%) [70].

The co-circulation and co-infection of these arboviruses has particular importance when one considers that diseases such as Guillain-Barré syndrome (GBS), which has an autoimmune component, are usually triggered by an infectious process [71]. GBS has been associated with infection by DENV [72], CHIKV [73], and ZIKV [74] in our country, which could increase the risk of developing the syndrome, the mortality rates, and epidemiological complications. Notably, recent publications with clinical and histopathologic evidence of patients co-infected with DENV/CHIKV demonstrate that there may be an association between mortality and co-infection with these arboviruses [75]; moreover, recent articles have demonstrated, both in vitro and in vivo, antibody-dependent potentiation in patients with anti-DENV antibodies who acquire the ZIKV infection [76, 77] or vice versa [78].

## Conclussion

Bearing in mind the epidemiological and clinical significance of co-circulation and co-infections, the possible outcomes arising from this situation should be studied in depth, particularly in regions in which the co-circulation of the three agents continues. These regions are significant public health concerns because the majority of the regions currently suffering ZIKV epidemics are also endemic for DENV and possibly for CHIKV, as in Colombia.

## Additional files

Additional file 1: Figure S1. Phylogenetic analysis of DENV-1 circulating strain in Villa del Rosario (2015). The evolutionary history was deduced by using the Neighbor-Joining protocol for gene pr-M protein, using a fragment of 431 nucleotides. (DOCX 313 kb)

Additional file 2: Figure S2. Phylogenetic analysis of the DENV-2 circulating strain in Villa del Rosario. The evolutionary history was deduced by using the Neighbor-Joining protocol for gene pr-M protein, using a fragment of 429 nucleotides. (DOCX 414 kb)

Additional file 3: Figure S3. Phylogenetic analysis of DENV-3 strains isolated in Villa del Rosario. The evolutionary history was deduced by using the Neighbor-Joining protocol for gene pr-M protein, using a fragment of 430 nucleotides. (DOCX 392 kb)

Additional file 4: Figure S4. Phylogenetic analysis of CHIKV strains circulating in Villa del Rosario. The evolutionary history was deduced using the Neighbor-Joining protocol for Env protein gene, using a fragment of 326 nucleotides. (DOCX 418 kb)

## Abbreviations

CHIKV: Chikungunya Virus
DENV: Dengue Virus
PBS: Phosphate-Buffered Saline
WHO: World Health Organization
ZIKV: Zika Virus

## References

[1] Wilder-Smith A, Byass P (2016). The elusive global burden of dengue. Lancet Infect Dis.

[2] Organization WH, Research SPF, Diseases TiT, diseases WHODoCoNT, Epidemic WHO, Alert P: Dengue: guidelines for diagnosis, treatment, prevention and control: World health Organization; 2009.

[3] Gubler DJ (2011). Dengue, urbanization and globalization: the unholy trinity of the 21st century. Trop Med Health.

[4] Weaver SC (2014). Arrival of chikungunya virus in the new world: prospects for spread and impact on public health. PLoS Negl Trop Dis.

[5] Musso D, Cao-Lormeau VM, Gubler DJ (2015). Zika virus: following the path of dengue and chikungunya?. Lancet.

[6] Rodenhuis-Zybert IA, Wilschut J, Smit JM (2010). Dengue virus life cycle: viral and host factors modulating infectivity. Cell Mol Life Sci.

[7] Gutierrez G, Gresh L, Perez MA, Elizondo D, Aviles W, Kuan G, Balmaseda A, Harris E (2013). Evaluation of the diagnostic utility of the traditional and revised WHO dengue case definitions. PLoS Negl Trop Dis.

[8] Musso D, Gubler DJ (2016). Zika virus. Clin Microbiol Rev.

[9] Omarjee R, Prat C, Flusin O, Boucau S, Tenebray B, Merle O, Huc-Anais P, Cassadou S, Leparc-Goffart I (2014). Importance of case definition to monitor ongoing outbreak of chikungunya virus on a background of actively circulating dengue virus, St Martin, December 2013 to January 2014. Euro Surveill.

[10] Furuya-Kanamori L, Liang S, Milinovich G, RJS M, ACA C, Hu W, Brasil P, Frentiu FD, Dunning R, Yakob L (2016). Co-distribution and co-infection of chikungunya and dengue viruses. BMC Infect Dis.

[11] Soumahoro MK, Gerardin P, Boelle PY, Perrau J, Fianu A, Pouchot J, Malvy D, Flahault A, Favier F, Hanslik T (2009). Impact of chikungunya virus infection on health status and quality of life: a retrospective cohort study. PLoS One.

[12] Rodríguez-morales AJ, Cardona-ospina JA, Urbano-Garzón SF, Hurtado-Zapata JS (2016). Prevalence of post-chikungunya chronic inflammatory rheumatism: a systematic review and meta-analysis. Arthritis Care Res (Hoboken).

[13] Cao-Lormeau VM, Blake A, Mons S, Lastere S, Roche C, Vanhomwegen J, Dub T, Baudouin L, Teissier A, Larre P (2016). Guillain-Barre syndrome outbreak associated with Zika virus infection in French Polynesia: a case-control study. Lancet.

[14] Estofolete CF, Terzian ACB, Parreira R, Esteves A, Hardman L, Greque GV, Rahal P, Nogueira ML (2016). Clinical and laboratory profile of Zika virus infection in dengue suspected patients: a case series. J Clin Virol.

[15] Acevedo N, Waggoner J, Rodriguez M, Rivera L, Landivar J, Pinsky B, Zambrano H (2017). Zika virus, chikungunya virus, and dengue virus in cerebrospinal fluid from adults with neurological manifestations, Guayaquil, Ecuador. Front Microbiol.

[16] Villar LA, Rojas DP, Besada-Lombana S, Sarti E (2015). Epidemiological trends of dengue disease in Colombia (2000-2011): a systematic review. PLoS Negl Trop Dis.

[17] Martinez-Gutierrez M, Ruiz-Saenz J (2014). Chikungunya in Colombia: chronicle of an epidemic foretold. Curare.

[18] Mattar S, Miranda J, Pinzon H, Tique V, Bolanos A, Aponte J, Arrieta G, Gonzalez M, Barrios K, Contreras H (2015). Outbreak of chikungunya virus in the north Caribbean area of Colombia: clinical presentation and phylogenetic analysis. J Infect Dev Ctries.

[19] Pacheco O, Beltran M, Nelson CA, Valencia D, Tolosa N, Farr SL, Padilla AV, Tong VT, Cuevas EL, Espinosa-Bode A, et al. Zika virus disease in Colombia - preliminary report. N Engl J Med. 2016; 10.1056/NEJMoa1604037 27305043

[20] Alarcon-Cruz AP, Prieto-Suarez E (2016). Description of the process of preparation and response of local health authorities facing the introduction of the chikungunya virus in Colombia, 2014. Rev Salud Publica (Bogota).

[21] WHO P-: Alerta epidemiológica: Fiebre por Chikungunya y Dengue en las Américas. 29 de Agosto 2014. In: Organización Panamericana de la Salud/Organización Mundial de la Salud. http://www.paho.org/hq/index.php?option=com_docman&task=doc_download&Itemid=&gid=27051&lang=es: Organización Panamericana de la Salud/Organización Mundial de la Salud; 2014.

[22] WHO P-: Infección por virus Zika 7 de mayo de 2015. In: Organización Panamericana de la Salud/Organización Mundial de la Salud*.* http://www.paho.org/hq/index.php?option=com_docman&task=doc_view&Itemid=270&gid=30076&lang=es: Organización Panamericana de la Salud/Organización Mundial de la Salud; 2015.

[23] Villamil-Gomez WE, Gonzalez-Camargo O, Rodriguez-Ayubi J, Zapata-Serpa D, Rodriguez-Morales AJ (2016). Dengue, chikungunya and Zika co-infection in a patient from Colombia. J Infect Public Health.

[24] Sardi SI, Somasekar S, Naccache SN, Bandeira AC, Tauro LB, Campos GS, Chiu CY (2016). Co-infections from Zika and chikungunya virus in Bahia, Brazil identified by metagenomic next-generation sequencing. J Clin Microbiol.

[25] Villamil-Gomez WE, Rodriguez-Morales AJ, Uribe-Garcia AM, Gonzalez-Arismendy E, Castellanos JE, Calvo EP, Alvarez-Mon M, Musso D (2016). Zika, dengue, and chikungunya co-infection in a pregnant woman from Colombia. Int J Infect Dis.

[26] Pessôa R, Patriota JV, Md L d S, Felix AC, Mamede N, Sanabani SS (2016). Investigation into an outbreak of dengue-like illness in Pernambuco, Brazil, revealed a Cocirculation of Zika, chikungunya, and dengue virus type 1. Medicine (Baltimore).

[27] Chien LJ, Liao TL, Shu PY, Huang JH, Gubler DJ, Chang GJ (2006). Development of real-time reverse transcriptase PCR assays to detect and serotype dengue viruses. J Clin Microbiol.

[28] Waggoner JJ, Abeynayake J, Sahoo MK, Gresh L, Tellez Y, Gonzalez K, Ballesteros G, Pierro AM, Gaibani P, Guo FP (2013). Single-reaction, multiplex, real-time rt-PCR for the detection, quantitation, and serotyping of dengue viruses. PLoS Negl Trop Dis.

[29] Pfeffer M, Linssen B, Parker MD, Kinney RM (2002). Specific detection of chikungunya virus using a RT-PCR/nested PCR combination. J Vet Med B Infect Dis Vet Public Health.

[30] Gomez-Calderon C, Mesa-Castro C, Robledo S, Gomez S, Bolivar-Avila S, Diaz-Castillo F, Martinez-Gutierrez M (2017). Antiviral effect of compounds derived from the seeds of Mammea Americana and Tabernaemontana Cymosa on dengue and chikungunya virus infections. BMC Complement Altern Med.

[31] Lanciotti RS, Kosoy OL, Laven JJ, Velez JO, Lambert AJ, Johnson AJ, Stanfield SM, Duffy MR (2008). Genetic and serologic properties of Zika virus associated with an epidemic, yap state, Micronesia, 2007. Emerg Infect Dis.

[32] Waggoner JJ, Pinsky BA (2016). Zika virus: diagnostics for an emerging pandemic threat. J Clin Microbiol.

[33] L'Huillier AG, Lombos E, Tang E, Perusini S, Eshaghi A, Nagra S, Frantz C, Olsha R, Kristjanson E, Dimitrova K (2017). Evaluation of Altona diagnostics RealStar Zika virus reverse transcription-PCR test kit for Zika virus PCR testing. J Clin Microbiol.

[34] Paiva MHS, Guedes DRD, Leal WS, Ayres CFJ (2017). Sensitivity of RT-PCR method in samples shown to be positive for Zika virus by RT-qPCR in vector competence studies. Genet Mol Biol.

[35] Faye O, Faye O, Diallo D, Diallo M, Weidmann M, Sall AA (2013). Quantitative real-time PCR detection of Zika virus and evaluation with field-caught mosquitoes. Virol J.

[36] Naccache S, Theze J, Sardi SI, Somasekar S, Greninger AL, Bandeira AC, Campos GS, Tauro LB, Faria NR, Pybus OG (2016). Distinct Zika virus lineage in Salvador, Bahia, Brazil. Emerg Infect Dis.

[37] Diaz-Quijano F, Gonzalez-Rangel A, Gomez-Capacho A, Espindola-Gomez R, Martinez-Vega R, Villar-Centeno L (2008). Rrainfall and acute febrile syndrome in a dengue-endemic area. Rev Salud Publica (Bogota).

[38] Rodríguez-Villamarín FR, Prieto-Suarez E, Escandón PL, de la Hoz Restrepo F (2014). Leptospirosis percentage and related factors in patients having a presumptive diagnosis of dengue, 2010-2012. Rev Salud Publica (Bogota).

[39] Gomez Arcila V, Arroyo Salgado BJ, Bello Espinosa AA, Rodriguez Escobar Z, Polo Andrade ER (2015). Microbiological diagnosis compatible with Anaplasma sp. in a patient suffering from febrile syndrome. Rev Argent Microbiol.

[40] Mattar S, Garzon D, Tadeu L, Faccini-Martinez AA, Mills JN (2014). Serological diagnosis of hantavirus pulmonary syndrome in a febrile patient in Colombia. Int J Infect Dis.

[41] Arroyave E, Londono AF, Quintero JC, Agudelo-Florez P, Arboleda M, Diaz FJ, Rodas JD (2013). Etiology and epidemiological characterization of non-malarial febrile syndrome in three municipalities of Uraba (Antioquia), Colombia. Biomedica.

[42] Romero-Vega L, Pacheco O, de la Hoz-Restrepo F, Diaz-Quijano FA (2014). Evaluation of dengue fever reports during an epidemic, Colombia. Rev Saude Publica.

[43] Ocazionez RE, Gómez SY, Cortés FM (2007). Dengue hemorrhagic fever serotype and infection pattern in a Colombian endemic area. Rev Salud Publica (Bogota).

[44] Alvis-Guzman N, Rodriguez-Barreto H, Mattar-Velilla S (2015). Dengue in an area of the Colombian Caribbean. Colomb Med (Cali).

[45] Ramachandran VG, Das S, Roy P, Hada V, Mogha NS (2016). Chikungunya: a reemerging infection spreading during 2010 dengue fever outbreak in National Capital Region of India. Virusdisease.

[46] Singh P, Mittal V, Rizvi MMA, Chhabra M, Sharma P, Rawat DS, Bhattacharya D, Chauhan LS, Rai A (2012). The first dominant co-circulation of both dengue and chikungunya viruses during the post-monsoon period of 2010 in Delhi, India. Epidemiol Infect.

[47] Carabali M, Lim JK, Velez DC, Trujillo A, Egurrola J, Lee KS, Kaufman JS, DaSilva LJ, Velez ID, Osorio JE (2017). Dengue virus serological prevalence and seroconversion rates in children and adults in Medellin, Colombia: implications for vaccine introduction. Int J Infect Dis.

[48] Roth A, Mercier A, Lepers C, Hoy D, Duituturaga S, Benyon E, Guillaumot L, Souares Y (2014). Concurrent outbreaks of dengue, chikungunya and Zika virus infections - an unprecedented epidemic wave of mosquito-borne viruses in the Pacific 2012-2014. Euro Surveill.

[49] Cardona-Ospina JA, Villamil-Gomez WE, Jimenez-Canizales CE, Castaneda-Hernandez DM, Rodriguez-Morales AJ (2015). Estimating the burden of disease and the economic cost attributable to chikungunya, Colombia, 2014. Trans R Soc Trop Med Hyg.

[50] Teixeira MG, Siqueira JB, Ferreira GL, Bricks L, Joint G (2013). Epidemiological trends of dengue disease in Brazil (2000-2010): a systematic literature search and analysis. PLoS Negl Trop Dis.

[51] San Martin JL, Brathwaite O, Zambrano B, Solorzano JO, Bouckenooghe A, Dayan GH, Guzman MG (2010). The epidemiology of dengue in the americas over the last three decades: a worrisome reality. Am J Trop Med Hyg.

[52] Berger S: Chikungunya and Zika: Global Status: 2016 Edition. In*.* Edited by Berger S. Los Angeles, California, USA.: GIDEON Informatics, Inc.; 2016: 121.

[53] Dirlikov E. Update: ongoing Zika virus transmission—Puerto Rico, November 1, 2015–April 14, 2016. MMWR Morb Mortal Wkly Rep. 2016;65 10.15585/mmwr.mm6517e2 27149205

[54] Morrison AC, Zielinski-Gutierrez E, Scott TW, Rosenberg R (2008). Defining challenges and proposing solutions for control of the virus vector Aedes Aegypti. PLoS Med.

[55] Mlakar J, Korva M, Tul N, Popovic M, Poljsak-Prijatelj M, Mraz J, Kolenc M, Resman Rus K, Vesnaver Vipotnik T, Fabjan Vodusek V (2016). Zika virus associated with microcephaly. N Engl J Med.

[56] Valentine G, Marquez L, Pammi M (2016). Zika virus-associated microcephaly and eye lesions in the newborn. J Pediatric Infect Dis Soc.

[57] Epidemiological alert: neurological syndrome, congenital malformations, and Zika virus infection: implications for public health in the Americas [http://www.paho.org/hq/index.php?option=com_docman&task=doc_view&Itemid=270&gid=32405&lang=em].

[58] Weaver SC, Vasilakis N (2009). Molecular evolution of dengue viruses: contributions of phylogenetics to understanding the history and epidemiology of the preeminent arboviral disease. Infect Genet Evol.

[59] Ocazionez-Jiménez RE, Ortiz-Báez AS, Gómez-Rangel SY, Miranda-Esquivel DR (2013). Dengue virus serotype 1 (DENV-1) from Colombia: its contribution to dengue occurrence in Santander. Biomedica.

[60] Gómez S, Villabona-Arenas C, Torres FA, Miranda-Esquivel D, Ocazionez RE (2008). Dengue virus serotype-3 (genotype III) from Colombia: perspective of its pathogenic potential. Dengue Bulletin.

[61] Laiton-Donato K, Usme-Ciro JA, Rico A, Pardo L, Martínez C, Salas D, Ardila S, Páez A (2015). Phylogenetic analysis of chikungunya virus in Colombia: evidence of purifying selection in the E1 gene. Biomedica.

[62] Rodas JD, Kautz T, Camacho E, Paternina L, Guzman H, Diaz FJ, Blanco P, Tesh R, Weaver SC (2016). Genetic characterization of northwestern Colombian chikungunya virus strains from the 2014-2015 epidemic. Am J Trop Med Hyg.

[63] Diaz-Quinonez JA, Escobar-Escamilla N, Wong-Arambula C, Vazquez-Pichardo M, Torres-Longoria B, Lopez-Martinez I, Ruiz-Matus C, Kuri-Morales P, Ramirez-Gonzalez JE (2016). Asian genotype Zika virus detected in traveler returning to Mexico from Colombia, October 2015. Emerg Infect Dis.

[64] Adiga R (2016). Phylogenetic analysis of the NS5 gene of Zika virus. J Med Virol.

[65] Saswat T, Kumar A, Kumar S, Mamidi P, Muduli S, Debata NK, Pal NS, Pratheek BM, Chattopadhyay S, Chattopadhyay S (2015). High rates of co-infection of dengue and chikungunya virus in Odisha and Maharashtra, India during 2013. Infect Genet Evol.

[66] Salas-Benito JS, De Nova-Ocampo M (2015). Viral interference and persistence in mosquito-borne Flaviviruses. J Immunol Res.

[67] Drumond BP, Mondini A, Schmidt DJ, de Morais Bronzoni RV, Bosch I, Nogueira ML (2013). Circulation of different lineages of dengue virus 2, genotype American/Asian in Brazil: dynamics and molecular and phylogenetic characterization. PLoS One.

[68] Yamanaka A, Mulyatno KC, Susilowati H, Hendrianto E, Ginting AP, Sary DD, Rantam FA, Soegijanto S, Konishi E (2011). Displacement of the predominant dengue virus from type 2 to type 1 with a subsequent genotype shift from IV to I in Surabaya, Indonesia 2008-2010. PLoS One.

[69] Chahar HS, Bharaj P, Dar L, Guleria R, Kabra SK, Broor S (2009). Co-infections with chikungunya virus and dengue virus in Delhi, India. Emerg Infect Dis.

[70] Waggoner JJ, Gresh L, Vargas MJ, Ballesteros G, Tellez Y, Soda KJ, Sahoo MK, Nunez A, Balmaseda A, Harris E (2016). Viremia and clinical presentation in Nicaraguan patients infected with Zika virus, chikungunya virus, and dengue virus. Clin Infect Dis.

[71] Yuki N, Hartung HP (2012). Guillain-Barré syndrome. N Engl J Med.

[72] Soares CN, Cabral-Castro M, Oliveira C, Faria LC, Peralta JM, Freitas MR, Puccioni-Sohler M (2008). Oligosymptomatic dengue infection: a potential cause of Guillain Barre syndrome. Arq Neuropsiquiatr.

[73] Villamil-Gomez W, Silvera LA, Paez-Castellanos J, Rodriguez-Morales AJ (2016). Guillain-Barre syndrome after chikungunya infection: a case in Colombia. Enferm Infecc Microbiol Clin.

[74] Arias A, Torres-Tobar L, Hernandez G, Paipilla D, Palacios E, Torres Y, Duran J, Ugarte US, Ardila-sierra A, Castellanos G (2017). Guillain-Barre syndrome in patients with a recent history of Zika in Cucuta, Colombia: a descriptive case series of 19 patients from December 2015 to march 2016. J Crit Care.

[75] Mercado M, Acosta-Reyes J, Parra E, Pardo L, Rico A, Campo A, Navarro E, Viasus D. Clinical and histopathological features of fatal cases with dengue and chikungunya virus co-infection in Colombia, 2014 to 2015. Euro Surveill. 2016;21(22) 10.2807/1560-7917.ES.2016.21.22.30244 27277216

[76] Priyamvada L, Quicke KM, Hudson WH, Onlamoon N, Sewatanon J, Edupuganti S, Pattanapanyasat K, Chokephaibulkit K, Mulligan MJ, Wilson PC (2016). Human antibody responses after dengue virus infection are highly cross-reactive to Zika virus. Proc Natl Acad Sci U S A.

[77] Dejnirattisai W, Supasa P, Wongwiwat W, Rouvinski A, Barba-Spaeth G, Duangchinda T, Sakuntabhai A, Cao-Lormeau VM, Malasit P, Rey FA (2016). Dengue virus sero-cross-reactivity drives antibody-dependent enhancement of infection with zika virus. Nat Immunol.

[78] Kawiecki AB, Christofferson RC (2016). Zika virus-induced antibody response enhances dengue virus serotype 2 replication in vitro. J Infect Dis.

