# Supplementary figures and images for: Co-circulation and simultaneous co-infection of dengue, chikungunya, and zika viruses in patients with febrile syndrome at the Colombian-Venezuelan border
Source: BMC Infect Dis. 2018 Jan 30;18:61. doi: 10.1186/s12879-018-2976-1 (PMC5791178; doi:10.1186/s12879-018-2976-1)

**
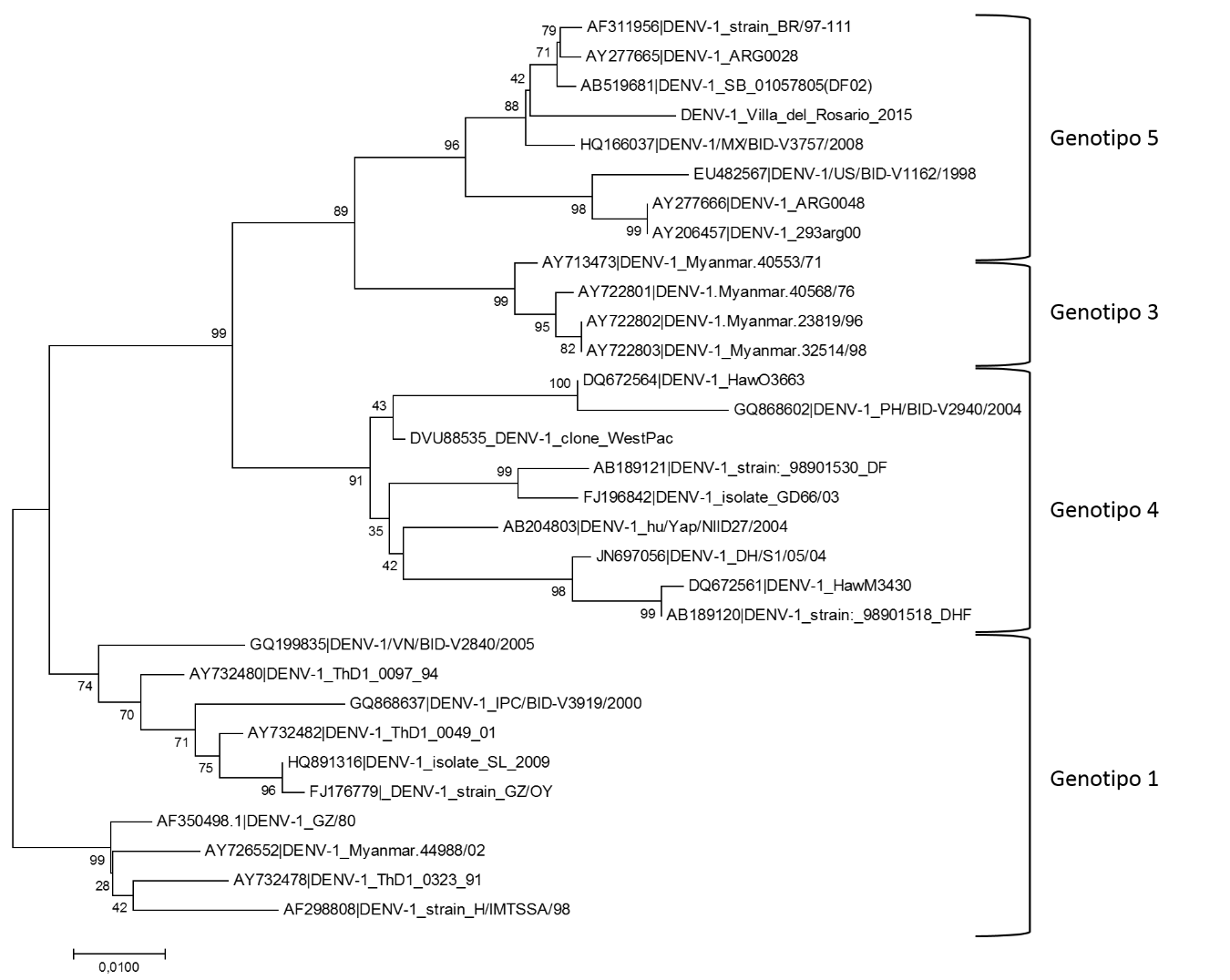
**

**Additional file 1: Figure S1.**

Supplement: Supplementary file 1 — Phylogenetic analysis of DENV-1 circulating strain in Villa del Rosario (2015). The evolutionary history was deduced by using the Neighbor-Joining protocol for gene pr-M protein, using a fragment of 431 nucleotides. (DOCX 313 kb) [file 12879_2018_2976_MOESM1_ESM.docx]

**
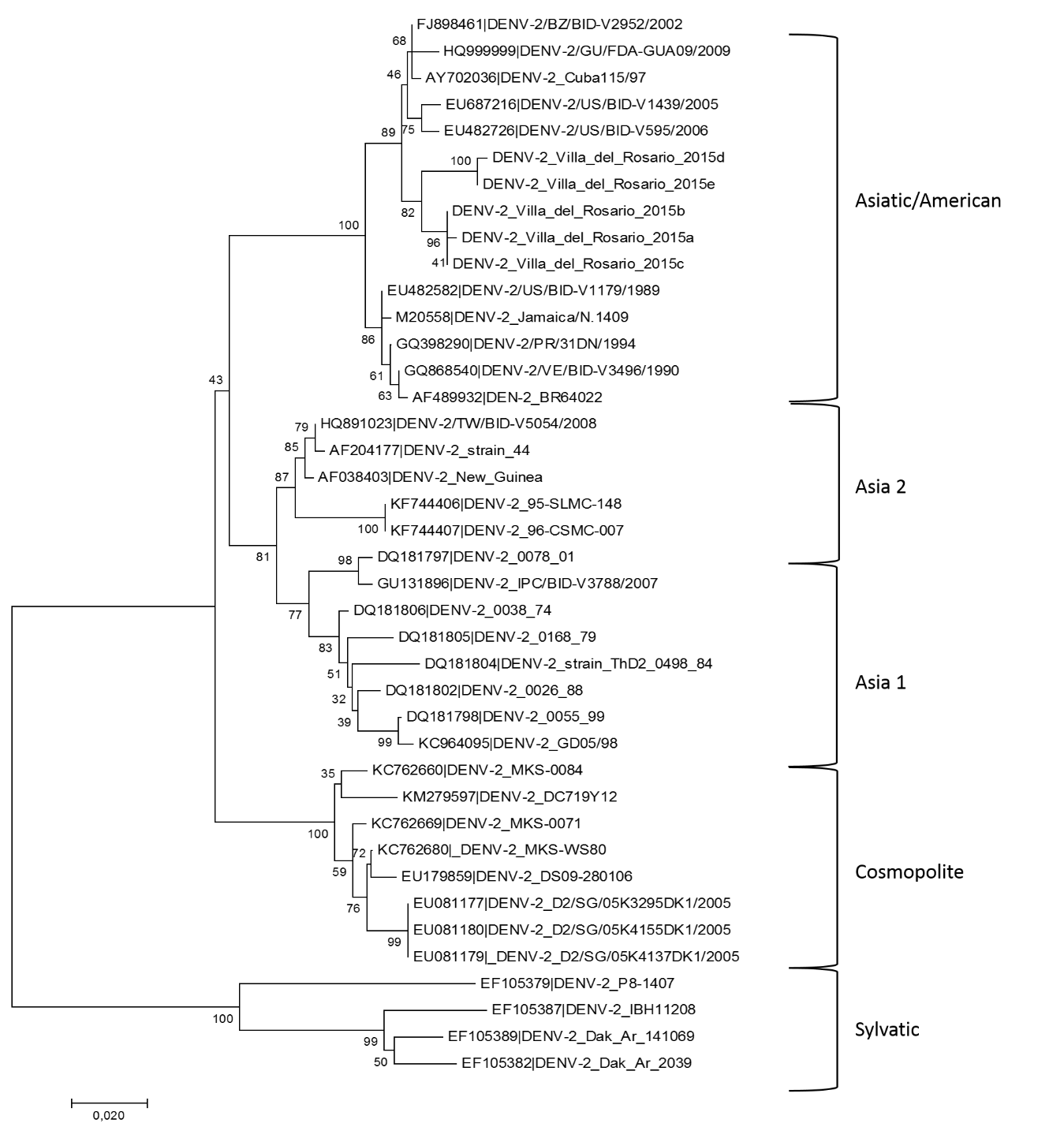
**

**Additional file 2: Figure S2.**

Supplement: Supplementary file 2 — Phylogenetic analysis of the DENV-2 circulating strain in Villa del Rosario. The evolutionary history was deduced by using the Neighbor-Joining protocol for gene pr-M protein, using a fragment of 429 nucleotides. (DOCX 414 kb) [file 12879_2018_2976_MOESM2_ESM.docx]

**
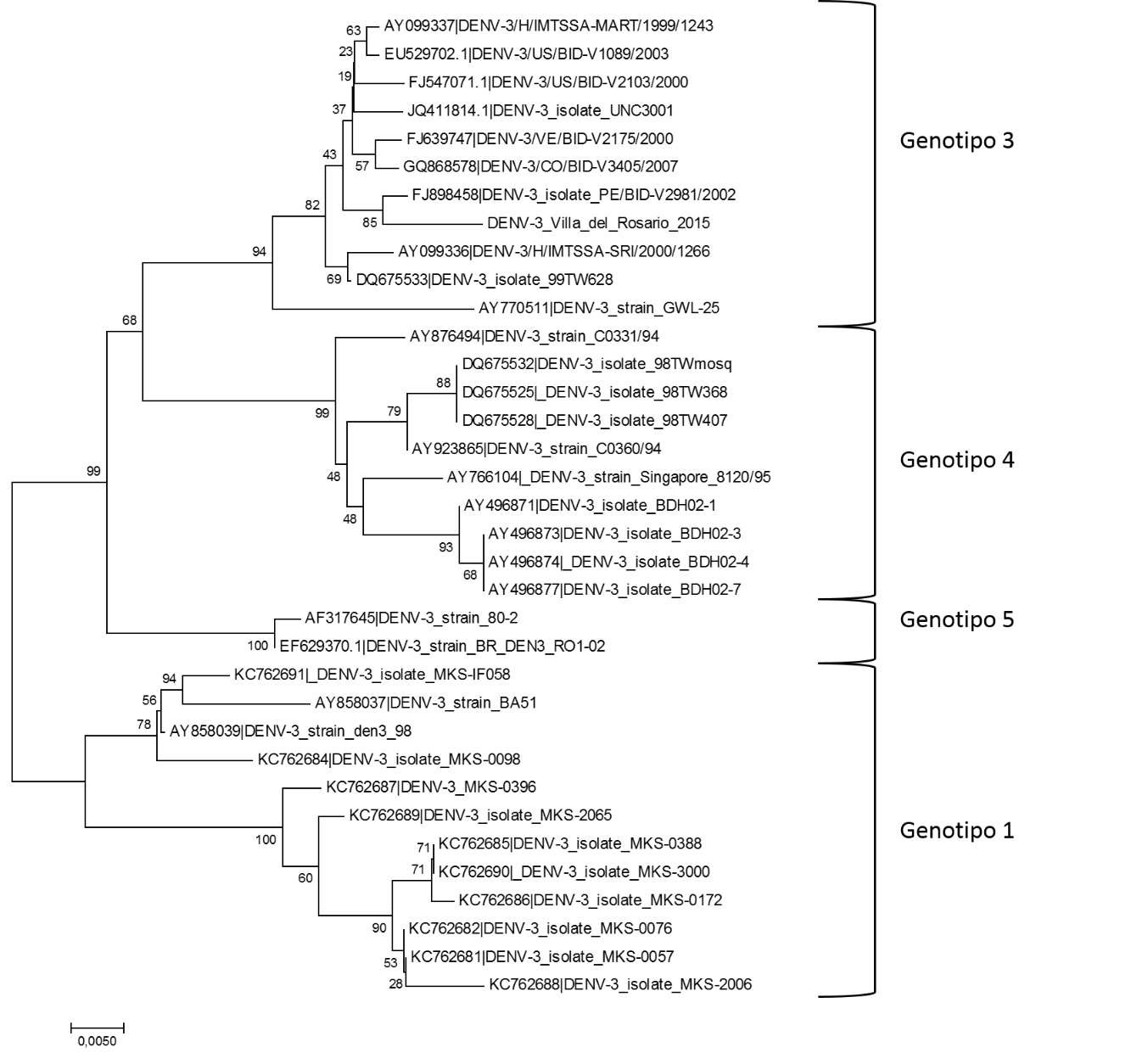
**

**Additional file 3: Figure S3.**

Supplement: Supplementary file 3 — Phylogenetic analysis of DENV-3 strains isolated in Villa del Rosario. The evolutionary history was deduced by using the Neighbor-Joining protocol for gene pr-M protein, using a fragment of 430 nucleotides. (DOCX 392 kb) [file 12879_2018_2976_MOESM3_ESM.docx]

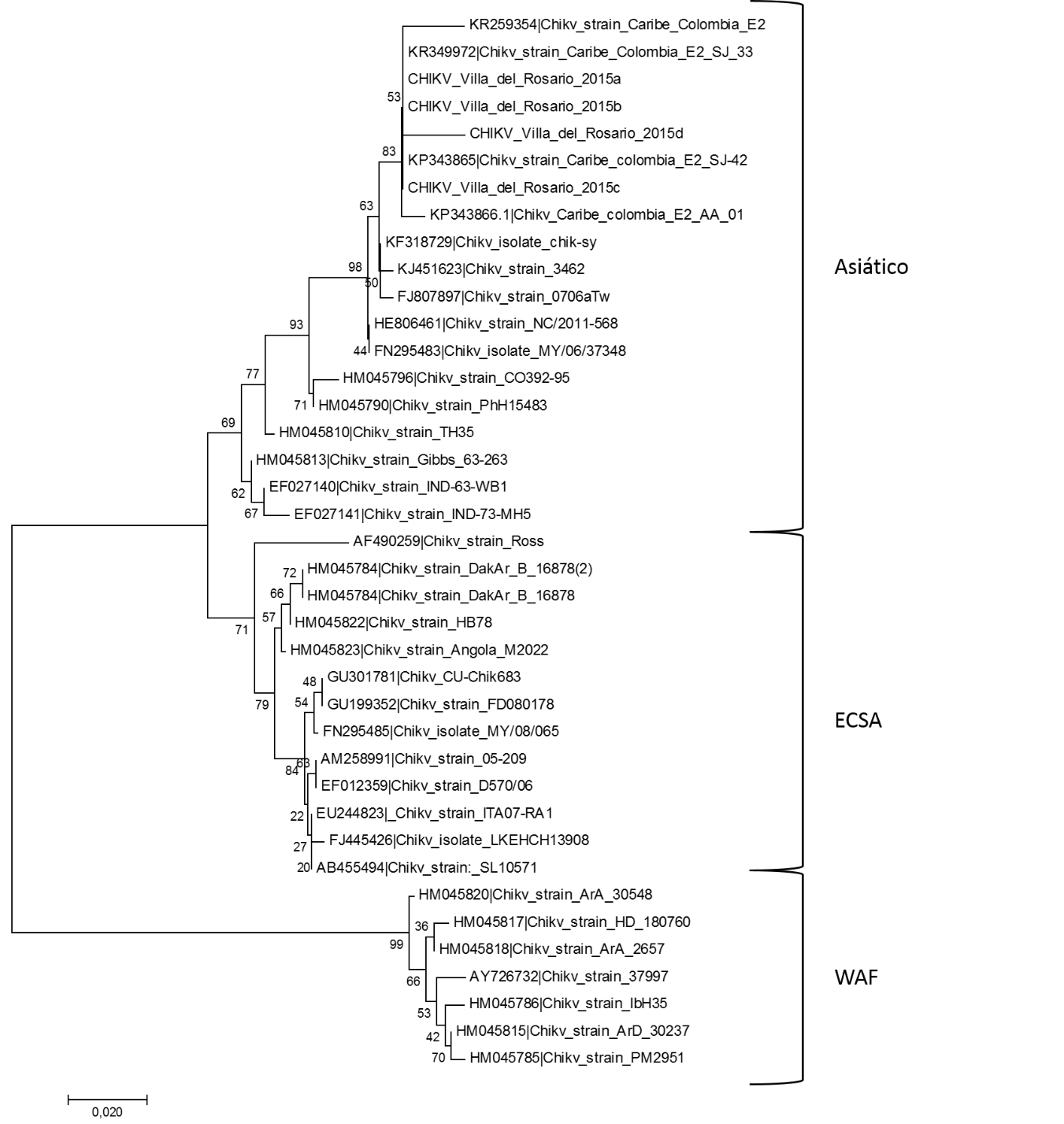


**Additional file 4: Figure S4.**

Supplement: Supplementary file 4 — Phylogenetic analysis of CHIKV strains circulating in Villa del Rosario. The evolutionary history was deduced using the Neighbor-Joining protocol for Env protein gene, using a fragment of 326 nucleotides. (DOCX 418 kb) [file 12879_2018_2976_MOESM4_ESM.docx]
